# Study of human rights violations faced by women who use drugs in Estonia

**DOI:** 10.1186/s12954-018-0259-1

**Published:** 2018-11-06

**Authors:** Arune Kontautaite, Daria Matyushina-Ocheret, Maria Plotko, Mikhail Golichenko, Mart Kalvet, Lena Antonova

**Affiliations:** 1 Eurasian Harm Reduction Association (EHRA), Verkiu St. 34b, 7 Fl., LT-09108 Vilnius, Lithuania; 2 Canadian HIV/AIDS Legal Network, 40 Bay Street, Suite 600, Toronto, Ontario M5R 2A7 Canada; 3 Estonian Association of People Who Use Psychotropic Substances (LUNEST), Pärnu Maantee 130-25, 11317 Tallinn, Estonia

**Keywords:** Women who use drugs, HIV, Human rights, Gender-based violence, Parental rights, Harm reduction, Gender-sensitive services, Drug policy, Estonia

## Abstract

**Background:**

Estonia continues to have the highest prevalence of HIV among people who inject drugs, and the highest overdose mortality, in the European Union. In August 2017, the Eurasian Harm Reduction Association (EHRA), the Canadian HIV/AIDS Legal Network (CHALN), and the Estonian Association of People Who Use Psychotropic Substances (LUNEST) conducted a study in Estonia to assess the situation regarding the human rights of women who use drugs and/or living with HIV.

**Methods:**

The research methodology, developed by EHRA and CHALN, comprised in-depth interviews with 38 drug-dependent women conducted between August 8 and 14, 2017, in Tallinn and Ida-Viru county. The interviews were transcribed, and 37 were analyzed using thematic content analysis.

**Results:**

The study has documented widespread violations of parental rights (removal of children because of their mother’s inability to cease drug use and barriers to regaining custody), violations of the right to health (the failure to provide quality drug and HIV treatment, and the disclosure of medical data, including HIV status and opioid substitution treatment (OST) records), the violation of labor rights due to drug use, arbitrary arrest, street drug testing, and violations of the right to a fair trial. A number of women have experienced repeated cases of gender-based violence but have had no access to psychosocial support, shelters, or other protection or rehabilitation measures.

**Conclusions:**

Our findings suggest that punitive drug laws and their enforcement practices, the lack of gender-specific drug treatment facilities, combined with stigma related to drugs and HIV, are the main drivers of systematic and serious violations of the human rights of women who use drugs or who are drug dependent. Stigma and human rights violations undermine Estonia’s efforts in HIV prevention, care, and treatment, and its overall efforts to respect, protect, and fulfill the right to health of women who use drugs or who are drug dependent. For these reasons, the Government of Estonia should address a variety of issues related to the protection of human rights of this vulnerable population group.

## Background

The situation of women who use drugs has been gaining importance in the discourse on HIV prevention and drug policy reform in recent years. Major international organizations, such as the United Nations (UN) [1], the European Union (EU) [2], the Council of Europe [3], civil society organizations [4], and academia have been paying increasing attention to the vulnerability of such women. Due to the stigma and social stereotypes related to drug use, women who use drugs are particularly vulnerable to domestic violence and they are regularly denied access to essential healthcare services and social support [4]. This is reinforced by repressive drug policies that allow for arbitrary detention and ill-treatment by law enforcement agencies, and thus contribute significantly to human rights violations of women who use drugs.

The recent shift from punitive and discriminatory drug policies toward health- and human rights-based drug control approaches is long overdue. In its 2014 statement, the United Nations Commission on Narcotic Drugs (CND) stressed the importance of drug policies consistent with human rights [5]. Member States reaffirmed this commitment during the United Nations General Assembly’s Special Session (UNGASS) on the *World Drug Problem* in 2016 [6]. They have also paid special attention to addressing the issue of non-discriminatory access by women to health, care, and social services in the context of drug use [6]. The EU bases its newest *Drug Strategy and Action Plan* on the fundamental values of the Union—i.e., respect for human dignity, liberty, democracy, equality, solidarity, the rule of law, and human rights [7, 8]. However, these and numerous other commitments and recommendations, remain mostly on paper, leaving the issue of human rights and gender-specific treatment services for women who use drugs primarily in the domain of research and expressions of concern by civil society and international organizations without truly being adopted by decision-makers of national governments.

The region of Central and Eastern Europe often lags behind in the implementation of international recommendations in the fields of HIV, drug policy, and human rights [9, 10]. Despite the scarcity of regional data based on research findings, reports clearly demonstrate the high relevance of the following regional issues: violence against women from intimate partners and the police [11], punitive drug policies resulting in violations of the rights of women who use drugs [12], and the lack of gender-specific treatment and rehabilitation services [13].

Estonia, the focus of the current study, is an Eastern European state that joined the EU in 2004 since when its domestic laws have undergone significant reform, although its drug laws remain predominantly punitive. Any act of illegal possession, or dealing in drugs, or possession not intended solely for personal use, is considered a criminal offense regardless of the type or amount of the illicit drug [14]. Activities such as the illegal manufacture, acquisition, theft or robbery, storage, transport, or delivery of narcotic drugs or psychotropic substances with the intent to supply are punishable by up to 3 years of imprisonment regardless of the quantity. Depending on the quantity of the drug and other aggravating circumstances (e.g., organized crime), a prison sentence of 6–20 years, or even life, in prison can be rendered [15].

Estonia reports more people, per capita, for drug crimes and offenses than the Russian Federation—one of the best-known global leaders in the war on drugs. In 2016, a total of 5,653 initial reports of drug-related criminal offenses and misdemeanors were recorded, which was more than in 2015; around 8 out of 10 of such offenses and misdemeanors were related to drug use and possession [16]. Thus, Estonia, with a population of 1,317,800 in 2016, registers 4.3 drug crimes and offenses per 1,000 population compared with 2.3 offenses and crimes per 1000 in the Russian Federation in 2016 [17].

HIV prevalence in Estonia is one of the highest in Europe: by December 31, 2017, a total of 9,492 cases of HIV had been reported [18]. Even though the annual number of newly diagnosed HIV cases attributed to injecting drug use is decreasing (30 in 2016, compared with 118 in 2010), the rate of new HIV infections in this population group remains one of the highest in Europe [16]. HIV prevalence among people who inject drugs is highest in Kohtla-Järve, a city in Ida-Viru county, (66%) and Tallinn (58%) [16], and women have represented 40% of all new HIV cases since 2013 [19].

Local and international studies focusing on injecting drug use have been carried out in Estonia in the past few years. For example, a recent study of people living with HIV who inject drugs, conducted in the cities of Kohtla-Järve in Estonia and St. Petersburg in the Russian Federation, concluded that experience of stigma is often associated with negative mental and physical health outcomes [20]. However, no research projects in Estonia have focused on the barriers faced by women in accessing harm reduction nor on the linkages between the discrimination of women who use drugs in healthcare settings and increased risk of HIV and violations of human rights. This information is of critical importance for policy making and in better planning of services.

Consequently, the current study aimed to shed light on the situation of Estonian women who use drugs in terms of discrimination they face in healthcare settings and the social support system and their vulnerability to violence and abuse and other violations of their social rights. The study was organized in 2017 as a partnership between international and local organizations—the Eurasian Harm Reduction Association (EHRA), the Canadian HIV/AIDS Legal Network (CHALN), and the Estonian Association of People Who Use Psychotropic Substances (LUNEST).

## Methods

The goal of the study was to explore violations of human rights experienced by women who use drugs in Estonia. The research question asked is “what influence does the current drug policy have on the discrimination of women who use drugs in health and social protection systems and the enjoyment of their human rights, including parental rights and access to healthcare?”

The research objectives included the following: (1) to document the violations of human rights of women who use drugs in Estonia, including discrimination in accessing health care and family and parental rights, (2) to analyze the connection between human rights violations and the criminalization of drug possession and stigmatization of women who use drugs in Estonia, (3) to explore the gender dimension of the documented human rights violations, and (4) to develop policy recommendations for the Government of Estonia on how to address the documented issues. The qualitative research methodology was developed by EHRA and CHALN. In-depth interviews were based on an interview guide which listed key topics but did not include a predefined list of questions.

Inclusion criteria comprised of females aged more than 18 years who had either Estonian or Russian as their native language and who were currently using drugs or had done so in the past. Those excluded from the study included females assessed as being in an unstable mental state.

Before data collection commenced, a seminar for the PWID community group, LUNEST, and other stakeholders was organized in Tallinn. During the seminar, the research goal, methodology, and ethical issues were discussed, as well as potential international and national-level advocacy following completion of the study. The recruitment process was organized by four representatives of LUNEST—one per geographic location: Tallinn and three cities in Ida-Viru county (Narva, Kohtla-Järve, and Jõhvi). The LUNEST representatives participated in the Tallinn seminar and were paid by the research project to inform the community about the research and to facilitate contact with potential interviewees who would likely provide rich information for documentation and analysis.

Participation in the study was voluntary; participants could refuse to answer questions at any point during the interview and withdraw the information already recorded. Each research participant was offered a supermarket gift certificate (the equivalent of EUR20) as an incentive.

Three international experts from EHRA conducted the interviews. Each interview was of approximately 40- to 60-min duration. Interviewers explained to participants the research goal, methods, voluntary participation, confidentiality, and data protection approach and informed them about the incentive, prior to each interview commencing. Each participant was given an information sheet about the research in their native language, and they were encouraged to keep it. Each participant was asked to read and sign an informed consent form in their native language. Participants’ names were not recorded, neither was their personal data that could be used for identification. Codes were used instead of names. Interviews were recorded, transcribed, and analyzed by thematic content analysis.

The methodology assigned an important role to the representatives of the local community in the study as follows: they were involved in planning the fieldwork and in recruiting research study participants, and they also acted as gatekeepers to ensure the linkage between researchers and women from the most vulnerable groups. Local activists were engaged in interpreting the research results and in developing a subsequent advocacy strategy.

The study methodology was not submitted for approval by an ethics committee because its primary purpose was to document human rights violations and analyze them. The study did not include any experiments on human subjects or undertake medical interventions. The research team reviewed Estonian legislation and consulted with community organizations and human rights activists on this matter. Since the research documented and analyzed self-reported data on drug use, HIV, former mental health issues, imprisonment, etc., the research team used a data protection protocol that ensured the secure storage of all hard copy and digital information related to the study. The research methodology ensured that the safety of the participants, their voluntary participation, and confidentiality issues were handled according to current international standards.

## Results

Interviews with 38 participants were conducted between August 8 and 14, 2017, in Tallinn, the capital of Estonia, and Ida-Viru county (Narva, Kohtla-Järve, and Jõhvi). One interview was excluded from further analysis due to the unstable mental state of the interviewee. All respondents are female and aged 26–46 years (mean age of 35 years).

**Table:** 

Age group	25–29	30–39	40–46
No. of respondents	5	27	5

All respondents have either Estonian citizenship or hold a permanent residence permit to live in Estonia. Thirty-three of the respondents speak Russian as their first language, and four are native Estonian speakers. Twenty-eight participants live in Ida-Viru while nine live in Tallinn. All participants have housing, including three who are provided with temporary social housing. Twelve participants have a professional education (equal to college level), 18 have full secondary education, and seven have not finished school. All participants are literate. Only eight participants are currently employed. Four respondents are married, and 11 are in a civil marriage. Nearly all (35) participants have children with seven of them having three or more children.

At the time of the interviews, all participants were currently using drugs or had used drugs in the past; 20 of them were receiving OST; and twenty-one respondents reported being HIV-positive and receiving antiretroviral therapy (ART). All information about drug dependence, HIV status, and other health-related issues were self-reported by the participants of the study.

Fourteen participants have a history of imprisonment, including a number of years spent in prison (up to 13 years). Two participants reported having received a large number of sentences (16 and 22 criminal court cases, respectively). All criminal cases concerned drug possession, drug-related theft, or other drug-related offenses.

All research participants received information about the study and signed consent forms. To ensure personal data protection and participant safety, names were coded and no references to their real names were included in the report. The interviews were fully transcribed and analyzed using thematic content analysis.

Violations of a number of human rights were recorded during the study, such as parental rights, the right to health, the disclosure of medical data, labor rights, and the right to a fair trial. A number of women reported repeated cases of gender-based violence.

There are three state agencies which, according to the drug laws, family law, and public health law, hold significant power with respect to women who use drugs: the police, child protection services, medical doctors, and public health authorities. These three agencies were reported in every interview as either preventing women from making healthy choices or directly violating their human rights. Local social workers in charge of child protection conduct home visits to families with reported drug use problems, and therefore, their opinions play an important role in making custody-related decisions. Some women reported that they had been pressured by child protection services to quit OST or attend long-term rehabilitation institutions to maintain custody of their children. In many instances, doctors and social workers have disclosed private medical information about the HIV status, or participation in drug treatment programs, to the family and employers of the interviewed women. Almost one third of study participants reported maltreatment by medical staff or were denied admission to a hospital because of their drug dependence or HIV status. Some study participants reported instances of police intimidation and coercion through the use of their children as leverage to gain information.

### Deprivation or restriction of parental rights

Twenty-five women reported the restriction or deprivation of child custody and/or parental rights because a parent was a drug user or drug dependent. Four women reported that they are, or have been, at risk of losing custody of their children because of their drug use, with the oldest cases of this nature occurring approximately 10 years ago.

In one particular case, a woman in Tallinn, aged 31, was deprived of her parental rights because the boyfriend with whom she lived was using drugs:

Participant: My boyfriend was put in jail. He is the father of my son. He spent seven months in prison. And Lastekaitse [the child protection service] came and said that I should leave him, otherwise the children will be taken away if I do not part ways with him. I began to say that I would not leave him because he was in jail. I do not want to do this. They insisted that I should do this.

Interviewer: Were you using [drugs] at the time?

Participant: No, I did not use [drugs] at that time. I had not even touched them. Then he [the boyfriend] came out of prison and started to use again...And then Lastekaitse came to me and said that they would assign two women who would come and check on me. They came twice, checked on me, and I did not use it [drugs] then, really. And they left, and my tests were clean. I even signed that they were clean. They left, and, a week later, Lastekaitse came and said, “You will receive papers stating that your children will be taken away because your tests show drugs.” And because of this, everything went downhill. I was not using before the children were taken away from me. I started using after they were taken away...

Interviewer: Why did they deprive you of your parental rights? I do not understand.

Participant: Because I have a boyfriend who is an addict. My civil husband is a drug addict. And I live with him...And they decided that it would be better for the children to be in an orphanage. I phoned my daughter’s dad in Finland and asked him to take our daughter...My son’s father was deprived of his parental rights because he did not go to court. Immediately he was automatically deprived...

In a number of cases, newborn babies were taken away from their mothers immediately after delivery and placed in a prenatal clinic in Tartu (130–170 km from their birthplace). The mothers were not allowed to participate in any decision-making related to the child’s health and were poorly informed about the child’s status. Despite having no legal justification, they were not permitted to take their children home from the hospital with them. Yet, in many cases, when mothers traveled to Tartu to see their babies, their travel expenses were not reimbursed.

Participant: With the second child, they just took him to an orphanage right from the hospital...Just because, in Maardu, we have a social worker who said, ‘We are taking him until the trial’.

Interviewer: Right after the delivery? Did you have to sign anything, any document, or did they just take him?Participant: They just took him. But I visited him in hospital.

(Aged 34, Tallinn)

Allegedly trying to protect the best interests of a child, child protection services visit parents with drug dependence to inspect the child’s living conditions. Unlike the police, child protection services conduct home inspections without any search warrant. They inspect kitchen refrigerators to see how much food is present and search wardrobes and talk to neighbors about the parents, often disclosing confidential information, such as their HIV status and/or other health conditions including, for example, drug dependence. Medical information about a parents’ health condition is often shared between the child protection and medical services.

The following is an account of a visit by social workers to the home of one participant:

Participant: The next day, I’m at home, and someone starts banging on my door. No one has ever banged this hard. And I realized that something bad was going to happen. I said, ‘Wait, wait, I’m coming already.’ I was on crutches, so it took me 5 minutes to get to the door...I opened the door, and there were these social workers. They immediately entered the room. They didn’t even try to discuss anything with me. They didn’t speak at all. Just, ‘That’s it — we are calling your mum. Look at yourself — we can’t leave the child with you.’...She went to the fridge, searched it — there was food in there. Then she moved to the children’s wardrobe with her shoes on, opened the wardrobe — there were clothes. She looked at the crib — it was new, everything was perfect. But, she said, ‘You are unworthy to be a mother, goodbye’.

Interviewer: Why?

Participant: Because I use drugs. Because my baby had been in hospital on methadone since she was born. We went to the social worker with my mom...And she [the social worker] told me to write a note saying that if I ever used [drugs] again they will take my children away from me and put them in an orphanage.

(Aged 28, Kohtla-Järve)

In several cases, women were forced to sign documents to show their “willingness” to have their parental rights restricted. In these cases, child protection services stipulated that if the woman refused to sign the papers to voluntarily relinquish their parental rights, their other children would be taken away.

Participant: And they said that either my child goes to an orphanage, or they leave him with his grandmother and grandfather if I write a refusal. Well, I wrote the refusal. Then, when I arrived at the prison, I understood what I had done. I submitted an appeal. Then there was a court hearing. In court, they took Sasha away from me, and my mother became his temporary guardian. I still had a long time left in prison. I got out of jail at the age of 26. They told my son that I was dead*.*

(Aged 35, Jõhvi)

Participant: There was a hearing to give my mother custody, and they told me that they would give my son to my mother if I waived my parental rights. It was my first child. I had to do it so that they [the social workers] would not take him to an orphanage. My mum took him. He spent two or three months with her, and she also had a little son of her own who was also two years old. He was hyperactive, a little bit troubled, and my mother couldn’t handle him. So, eventually, she gave my son to an orphanage.

(Aged 26, Tallinn)

Participant: Yes, a woman came to me and said, ‘If you don’t sign...’. At first she was just asking, trying to persuade me.

Interviewer: Who was this woman?

Participant: I don’t know, maybe she was some kind of social worker. To be honest, I don’t remember.

Interviewer: Did she give you some document to sign?

Participant: Yes, something like that. She wanted me to sign over my parental rights to his grandmother.

Interviewer: And the presumed social worker was coming every day?

Participant: Every day. Every day she would come and make me cry. She was following me to the bus stop...In the end she told me, ‘If you don’t do it now, your child will end up in an orphanage. I promise’.

Interviewer: And what did you do?

Participant: I signed over my rights.

(Aged 34, Tallinn)

A mother of three explained why she had lost custody of all her children under pressure from the child protection service:

Participant: I arrived at the child services and said that I wanted to see my child. This was the first time I went to them. I only had 10 days of sobriety — it is nothing, in general. They told me that I was eight months pregnant. ‘Let’s do it this way: you write a document saying that you give up your older children, Sasha and Dima, for a short time; and under these terms, we will let you keep your newborn baby.’...Then [after the youngest baby was taken away] I understood what they had done to me. And I was in a very terrible rage. I remember these six days as a rollercoaster, when I wanted to kill myself; I was ready to strangle myself due to all of this. And I understood that I had been fooled, that I gave up Sasha and Dima for half a year, so that they left Danka with me. And now I have limited rights with Danka.

(Aged 35, Jõhvi)

The interviewed women reported strong evidence of the child protection services either forcing them to stop OST, despite its importance to their health and stability, and to get clean under the threat of losing the custody of their children, or not allowing a child to stay with the other parent because this parent was a methadone patient.

Participant: Lastekaitse [the child protection service] then took my child away from me...I recently fought with them for 10 days when I gave birth to my second child. We were transferred to another hospital where she [the child] was given sedatives; they did not allow me to stay with her for 10 days. I was not allowed to see her until my tests were clean. But the hospital test showed drugs for 10 days. The drug will keep appearing in urine for 10 days. They thought I was using...Yes, they offered me rehabilitation, but in order to go there, I had to leave my daughter in an orphanage...I knew that I would never leave my daughter. I will not give her to anyone. And I said that I will not go to any rehabilitation because my child is dearer to me, because I won’t put her in an orphanage.

Interviewer: Why didn’t they let your husband take her? Why not give her to her father?

Participant: Because her father was also on methadone. We both had to go to rehabilitation to be clean even from methadone.

Interviewer: You are not allowed to be on methadone? Or did they have any other reasons?

Participant: No, he did not have any bad tests at all, only methadone, that’s it, so they wanted him to be clean from any substance.

(Aged 26, Tallinn)

Although a discriminatory provision for the deprivation of parental rights due to a parent’s drug dependence was repealed in 2009 [21], child protection services still consider drug use and drug dependence as reasons for restricting, or depriving, parental rights, assuming that any substance use puts a child in danger and, therefore, is contrary to the child’s interests, even when a parent takes medically prescribed methadone.

Participant: Then, after 15 days, when I came for my child, I was told, ‘We will not give you [the child]’ — because I am in a methadone program and come from a dysfunctional family, even though I have a two-room apartment in a good state of repair. And I felt so insulted...

(Aged 34, Narva)

To regain custody of their children, women have to attend an abstinence-based rehabilitation center for 12 months, immediately find a job (even though the Ida-Viru region has an unemployment rate that is double the Estonian average) and equip their apartments to a high standard. Moreover, there is not a single drug treatment center for women with children, or who are pregnant, in the whole of Estonia.

On a number of occasions, women have lost cases to restore custody of their child because of their low social status (having no regular job) or because of disabilities in their family. There are currently three known cases of women fighting to restore their parental rights and in need of quality legal and social support.

Participant: These social services have known me since I was a kid, and in court...I said, ‘You didn’t even give me the flat like you were supposed to* and now you are saying that I don’t have a place to live with my child. Give me the flat, I will get back my child, and everything will be fine.’ They said that I should choose between the flat and my son. They said that they will give me [the flat] if I waive my parental rights. Even these kinds of arguments...they said them in court.

(Aged 33, Tallinn)

* The respondent grew up in an orphanage and was entitled to a flat by law.

Participant: I was on methadone at that time and gave birth on methadone. I didn’t have any problems, you know. And then I moved to that new apartment; it was still in the process of being renovated. And they came. Again, only one room was unfinished; everything else was ready. Plus, all my clothes from the wardrobe were on my bed, and all this was considered to be a mess.

Interviewer: But the renovation was still in progress?

Participant: Yes. And they came with the police.

Interviewer: Social workers with the police?

Participant: Yes. And they looked at all this and decided that this place was not suitable for a child.

(Aged 34, Tallinn)

Participant: Then I was accused of not having a cradle for the second child...They put it all together, filed a court case. And the court decided that the city, Kohtla-Järve, would take custody of [my] children.

(Aged 44, Jõhvi)

None of the women interviewed received effective drug treatment before or during their pregnancy. While a number of participants were receiving OST before the child was removed, the quality of treatment, according to them, was low. Women did not receive social support, such as job placement or housing, that they needed to be able to provide quality living conditions for their children.

During criminal proceedings, accused mothers often have to sign papers to relinquish their parental rights under the threat that if they do not sign, their children will be sent to an orphanage and later to unknown foster parents, rather than to their grandparents, for example.

### Ill treatment by police and arbitrary detention

Child protection services often act together with the police, frequently conducting home inspections with police officers, allegedly to ensure the safety of social workers. In practice, the presence of the police inside or outside of a house serves to apply additional pressure on the parents.

Moreover, some study participants reported cases of coercion when the police used the fact that the women had children to intimidate or threaten them, trying to extract a confession or evidence against somebody else.

Participant: Vasya [name changed] is in Narva. He has just served five years for selling drugs...My girl was then going to the kindergarten; she was two and a half years old. I was walking on a street in the city and the police took me in... They wanted me to testify against Vasya. They said, ‘Well, what are we going to do with you? Your child is in the kindergarten. Who is going to pick her up? If you have someone to call, do it.’ She [the policewoman] started to play with me. I knew she could do it. I told her, ‘Write whatever you want, and I’ll sign it.’ And I signed that I had bought drugs in such and such quantities. And the fact that they [the police] were blackmailing me is true. Especially if a woman/a girl has a child, she will give evidence.

(Aged 32, Narva)

According to four women respondents, the police recognized them as being drug dependent and stopped them on the street to undergo a saliva drug test. According to these women and other study participants, if they refuse, they are taken to a police station and are forced to have a urine drug test through a catheter. This procedure is regulated by government Decree [22]. If the test is positive, the person must pay a fine and also reimburse the cost of the drug test—a total of more than EUR100—which is unaffordable to women who use drugs, many of whom live below the poverty line.

Participant: They made me take a test for alcohol because there were empty beer bottles on the kitchen table. My boyfriend drinks beer. Well, when he does not work, he drinks beer. It is his private affair. The test showed nothing. And then they made me take a drug test.

Interviewer: And you had not been breastfeeding the child for a year already?

Participant: No, he is on formula, not breastmilk.

Interviewer: How did they explain this? Why were they doing this?

Participant: Well, how...that they were obliged to do it.

Interviewer: Could you have refused?

Participant: This is an interesting question. If I refuse, they take away the child by default, in a moment. And later, nobody knows...I understand where it can lead. It is not a certainty that I will get him back; therefore, I really say, ‘Yes, I use and am afraid...’. Yes, I was threatened that the child would be taken away. And I agreed, of course, to the test. Yes, I played a fool. And the threat is that if they take me to a drug test lab, then they will use physical force. That is, they will take urine with a catheter.

(Aged 34, Kohtla-Järve)

The use of urinary catheters poses significant risk of infection of the urethra, bladder, and kidney [23]. Depending on the circumstances, forced urine tests with the use of urinary catheters can also be qualified as torture or a form of cruel, inhuman, or degrading treatment or punishment.

The reason for this policing practice is that people who use drugs are well known to the police—not due to any specific behavior or suspicious activity. This type of random drug testing constitutes arbitrary arrest and has severe consequences for women who use drugs, making them even more vulnerable to losing custody of their children. As a result, women lose confidence in state services. This lack of trust represents a barrier to drug and HIV prevention, treatment, and care, as well as to effective social reintegration for drug-dependent women.

Participant: A police car pulls up, and they say, ‘Your documents’. Sure thing. ‘Sit down, please. You will take a drugs test’. Why, what is it? ‘Well, here, you have a reputation for being an addict’.

Interviewer: And they take you away for a test?

Participant: No, right there, in the car. If you refuse, then they take you [to the station]. And they did the test once and it didn’t show anything, so they did it a second time and I had nothing.

(Aged 44, Jõhvi)

### Stigma and discrimination as obstacles to quality healthcare

Despite the HIV treatment guidelines of 2013 that recommends the initiation of HIV treatment at a Cluster of Differentiation (CD4) count of > 500, most respondents noted that their HIV treatment was delayed, leading to severe health conditions, lower treatment efficiency, and a higher risk of HIV transmission to their partners. Research studies, including those by the World Health Organization, demonstrate that people who use drugs have low access to HIV testing and ART, and drug treatment, including OST, is poorly connected with HIV services [24, 25].

The women reported that they did not want to get tested for HIV or start ART because of the stigma associated with the condition. In the following quote, the respondent explains why she could not undertake measures to prevent the transmission of HIV from mother to child (PMTCT):

Participant: I didn’t go to the maternity clinic only because I have a disease [HIV]...My mother worked in a hospital at that time. Once they learned that I had hepatitis, they submitted me to all the tests. Had they learned that I had HIV, they would have thrown me out immediately. This happens very fast here. They would find any pretext. That’s why I did not want to go [to have PMTCT].

(Aged 34, Kohtla-Järve)

During the interviews, 11 cases were reported of women being denied admission to hospital, or being improperly cared for, because of their drug dependence or HIV status.

Participant: I was given a depression assessment test. The test showed 10 out of 10 points, so they told the father of my child to go get my clothes immediately because I would be staying there [at the psychiatric clinic]. They told me they would admit me. They tried to find out the reasons for my depression but I refused to talk. The next 10 minutes went like this: they opened their computer, saw what pills I was taking, and then it became clear to them that I’m actually a drug addict. So they told us that they don’t admit drug addicts and when the father of my child asked what we should do, they told us to turn somewhere else. [In order to be admitted there] I would first have to get rid of my drug problem. The father of my child asked, ‘But you just said that she’s at risk of suicide?’ And they told him that we would be lucky to find help before that happens. And then we left.

(Aged 29, Tallinn)

Interviewer: Did you try to kill yourself?

Participant: Yes. It was before my first pregnancy. It was drugs. I knew that this was the end, that it is almost impossible to stop using this drug*.*

Interviewer: That’s why you cut your veins? And then you were admitted to the psychiatric hospital?

Participant: Yes. I spent one day and slept it off. They observed me and saw that I’m a normal, reasonable person. I talked with them like I’m talking with you now. And they let me go home.

Interviewer: One day? No psychiatrist?

Participant: No. One or two days, I don’t remember. I first swallowed the pills and then cut my veins.

(Aged 26, Tallinn)

Interviewer: This is a very sensitive topic. But these three pregnancies you had before...they told you that you had to have an abortion?

Participant: No, those were just miscarriages...

Interviewer: What did the doctor say to you when it happened?

Participant: He said that HIV ate it.

Interviewer: HIV?

Participant: Yes, HIV ate my baby. In hospital during labor, the doctor who helped me to deliver forced me to put on a mask. It was already hard to breath. They told me to stop panting and to put on the mask so I wouldn’t spit my HIV on them.

(Aged 34, Narva)

### Disclosure of medical information

The stigma associated with HIV or drug use is further heightened when private medical information, such as a person’s HIV status, is disclosed to their relatives and employers, or even in the workplaces of relatives and partners. According to participant accounts, the problem of HIV disclosure is less acute now than it was before, while the issue of unlawful disclosure of data related to drug dependence continues to be an issue. Altogether, five respondents reported disclosure of their private medical data. In these cases, medical professionals or child protection services acted as if they wanted to protect the public from HIV by sharing information about HIV-positive clients.

Participant: Our parents went for a visit. And this doctor lives in the same building where our parents went. We went with my husband to meet them. Afterwards, this doctor met with a woman whom my parents were visiting and told her, ‘You must wipe all the [door] handles after they’ve left and also disinfect all the buttons in the elevator’.

Interviewer: Did these people tell you this?

Participant: Yes. These friends told me that this doctor said so when they met her.

Interviewer: And does she still work in a hospital or in a polyclinic?

Participant: Yes, she still works in a polyclinic.

Interviewer: Did you try to tell anyone about this? The management at the clinic?

Participant: No, it’s useless. She is an Estonian...and she is the chief psychiatrist.

Interviewer: And these friends, they knew about your status?

Participant: Oh, no. That’s when I went to her for methadone. She wrote out [a prescription] for pills for me. I have not seen her since.

Interviewer: So she knew about you because you went to her?

Participant: Yes.

(Aged 34, Narva)

According to the women interviewed, child protection services can proactively contact family members or employers of OST patient to inform them that they are receiving treatment. The main reason for such behavior is a misunderstanding of OST. Study participants reported that child protection services stigmatize OST patients, wrongly believing that OST is no better than using street drugs.

Participant: One day my mother and my partner’s mother called us and told us to immediately come to a family meeting. We went, and, you know, I always lied to my mother that everything was fine. And these social workers, even though they are not allowed to talk about me being in the methadone program, my dose there, they told our parents everything: our dose, what our drug tests show, whether we use [drugs] or not.

(Aged 28, Kohtla-Järve)

### Violations of labor rights

The majority of women who participated in the study were unemployed, which, in turn, decreases their chances of social reintegration and, given current juridical practice, limits their ability to regain custody of their children. The main reason for such unemployment among women participants is the widespread disclosure of their HIV and drug dependence status, with six respondents reporting violations of their labor rights.

Participant: I had a job at a sewing factory. I felt ill. I fainted. I had a nosebleed. Well, they called an ambulance and they asked me if I took any pills or something, so that they could make an injection. Yes, I said that I take pills regularly. I just told the nurse that I take pills. Well, the following day I was asked to leave of my own free will.

Interviewer: And how did they explain this?

Participant: Well, so not to blow it out of proportion, ‘Since you are HIV-positive, we do not want [you] near sewing equipment, needles. We will not tell anybody anything, but at the same time you will write a resignation letter’.

Interviewer: And how did they find out?

Participant: Well, the nurse told them.

Interviewer: That is, the nurse from the ambulance told the authorities?

Participant: Yes.

(Aged 34, Narva)

Employers quite often receive confidential medical information directly from doctors or the staff of OST clinics and child protection services.

Participant: When I was just employed, starting work, I went to the child protection services and put my job contract on the table. I go to work the following day and I am called in by the owner. And he says, ‘Vika [name changed], I received a call today and they said that you have problems with drugs’.

(Aged 35, Jõhvi)

### Gender-based violence

Nine out of the 37 respondents had experienced repeated cases of violence by their intimate partners that often required medical attention. Most of these women did not trust the police or social services to help them in such cases.

Participant: When I was 13, I sort of started messing around. At first, my skull was kind of broken and I was in a coma for two days. Then I was raped when I was 14. I ran away from home. I lived on the streets for half a year.

(Aged 35, Jõhvi)

None of the women who participated in the study had heard about special services designed to help victims of domestic violence such as shelters, case management, or individual or group therapy. Old and current cases identified by the study demonstrate that no positive shift has taken place, and it appears that the police are ill-equipped to protect women who use drugs from gender-based violence.

Interviewer: Have you experienced violence against you?

Participant: Yes, the person with whom I lived used to beat me. He used to throw me out on the street so that I would go steal, then I could spend the night at his place.

Interviewer: And if you did not steal, you could not spend the night at his place?

Participant: Yes. I spent the whole day on the street.

Interviewer: Have you tried going somewhere, to some crisis center for women? Did you know of any?

Participant: No*.*

Interviewer: No? You did not know of such centers?

Participant: I did not know.

Interviewer: Did you take photos of the beatings? Did you go to the hospital?

Participant: No.

Interviewer: Why?

Participant: Because I believed that it was normal.

Interviewer: It is normal that he beats you?

Participant: Yes.

(Aged 33, Tallinn)

Police practices discourage women with children from contacting them if they experience gender-based violence. According to several documented cases, when women call the police in situations of aggressive behavior by their male partners, the police often inform child protection services and this may result in the loss of custody of a child. The police may also prosecute a woman for drug offenses instead of protecting her from gender-based violence.

### Lack of access to quality legal and social support services

The vulnerabilities of women who use drugs, or who are drug dependent, are not being addressed in Estonia. All respondents to this study reported very little, if any, social support, such as job placement or opportunities to improve their housing conditions to meet the standards required by the child protection services. Rather, the child protection services used the lack of good quality living conditions and/or the lack of a permanent job as a reason for restricting or depriving women of their parental rights and/or by taking a child away from the parents.

Women who use drugs often face legal challenges such as police prosecution, legal proceedings related to the child protection services, and discrimination in labor and public health matters. Yet, there is very limited access by such women to free legal support services. Women report that such legal services related to cases of criminal prosecution are of very poor quality.

Participant: Public defender...I remember I had one...He didn’t even come to the meeting, just discussed it all over the phone with the policeman.

(Aged 28, Kohtla-Järve)

According to the women interviewed, the lawyers provided by the State more often than not do provide an insufficient legal defense and act rather as an extension of the police and child protection services, supporting the toughest measures against women who use drugs, especially depriving them of their parental rights.

Participant: Lastekaitse [the child protection service] provided me with some lawyer, but as far as I could see he was on their side. He didn’t want me to have my children back either. He also said that I have to have my own flat. I asked why it should be my own if I’m renting a place for more than a year. Why can’t I just continue doing that if it is my permanent residence? He said no, they won’t allow it. I don’t know what kind of lawyer they were.

Interviewer: What did he say?

Participant: He said that I should be deprived of my parental rights.

(Aged 31, Tallinn)

## Discussion

As in many countries, women who use drugs in Estonia face losing custody of their children with drug use becoming the predominant factor in the child custody decision-making process of state authorities [26, 27]. The risk of a child being taken away is reinforced by stigma and social stereotypes that a woman who uses drugs cannot be a good mother [3]. Child protection services play an important role in decision-making when separating women who use drugs from their children which has multiple consequences for the families, the key impact being trauma. The use of drugs and alcohol becomes an important way to decrease the pain of separation which is reinforced by heightened vulnerability through increased exposure to housing instability, intimate partner violence, and initiation of injection drug use and sex work [27]. The concerns of women in relation to losing custody of their children prevents them from seeking treatment and hinders their access to other drug use-related services [28].

Child protection services in Estonia, often acting in a similar manner to the police even though they are not bound to do so by any procedural rules, are playing a role in drug enforcement. The fear of child protection services quite often becomes one of the main obstacles for women in accessing effective drug treatment. As stated by some of the study participants, employees of the child protection service have pressured drug-dependent women to stop taking medically prescribed methadone under the threat of terminating their parental rights. Restricting a parent’s rights based on their participation in a drug treatment program contradicts the World Health Organization’s recommendations which state that OST is the most effective type of opioid dependence therapy [29]. Drug dependence treatment, including OST, is available in Estonia. However, the coverage of OST is low (< 20%) [30]. Women also report poor quality OST services in general and especially so for women with children or who work. The specific needs of such clients are not accommodated, and social support is almost non-existent.

Drug treatment in Estonia is organized in such a way that it is virtually impossible for women to combine it with work, as only two options are available to them: to spend 12 months at an in-patient rehabilitation center or to join an OST program. To spend a year at a rehabilitation center is not viable for most women undertaking temporary work as they cannot be absent for such a long period of time. It is especially difficult for women with children. OST is a better option for working parents. However, according to national guidelines, most clients have to attend clinics on a daily basis. Take-home options are very restricted even for clients who have to travel for an hour every day to take the medication. It is often impossible to combine such trips with a work schedule, especially considering the desire of OST clients to not disclose their health status to an employer. Such situation with the provision of drug treatment is typical for the region of Eastern Europe and Central Asia where drug treatment regulations lead to the violation of labor rights of patients [31].

According to the study participants, most drug treatment doctors in Estonia are ready to provide drug treatment for women before, during, and after pregnancy. However, being afraid of losing the custody of their newborn child, women who use drugs either do not inform their gynecologist about their drug use/dependence or inform them only after the child is born. Where mental health issues are established, psychiatric examinations are conducted without informed consent and with an apparent intention to use the psychiatric diagnosis along with the mother’s drug use to substantiate a case to deprive her of her parental rights, leaving her with no social or medical support.

Drug treatment and infectious disease doctors and child protection services disclose private medical information of women living with HIV and/or drug-dependent women to the police, members of the public, employers, and family members. By doing so, public officials are playing an important role in further stigmatizing and ostracizing these women who, out of fear, do not seek appropriate treatment. Instead of facilitating access to testing and treatment for HIV and other infectious diseases, state authorities have become a serious obstacle to such services. State authorities should consider providing support to women who use drugs through effective drug treatment, case management, vocational training, and job placement, instead of punishment.

Disclosure of one’s drug use history makes a person especially vulnerable for further discrimination and abuse in a country with a repressive drug policy. In Estonia, the consumption or possession of narcotic drugs or psychotropic substances in small quantities is punishable by a fine of up to EUR1200 or detention of up to 30 days; according to the Ministry of Justice of Estonia, the average fine for possession of cannabis in 2015 was EUR80, the average fine for possession of any other drugs was EUR100, and the average fine for possession of any and all drugs was EUR90 [32]. This is a large sum in Estonia, where the current minimum wage is EUR500 per month [33]. Any act of drug dealing or possession not intended solely for personal use is considered a criminal offense, regardless of the type and amount of illicit drug punished with years of imprisonment [14, 15].

As the study has demonstrated, women who use drugs are often subjected to monetary fines for drug use and possession. Paying such amounts, sometimes several times a year, places a serious burden on women, the majority of whom live below the poverty line. It appears that courts in Estonia do not meaningfully consider a woman’s ability to pay, and this entraps women who use drugs in a cycle of fines, debt, and incarceration. Child protection services, in their turn, press women to prove that they have “good living conditions” for children, but chances of this decrease with every fine administered for drug use/possession. Moreover, a prior history of unpaid fines limits opportunities for employment, with many such women preferring “informal employment”; otherwise, almost all of their earnings would be withdrawn to pay off the debt. This resembles the “criminalization of poverty” phenomenon in the USA whereby the system of bail, fees, and fines result in damaged credit scores and decreased opportunities to find employment and housing [34].

The situation is aggravated by the fact that, as the study has shown, women’s rights are being violated when police officers conduct forced street drug testing, especially when women are forced to undergo such tests using urinary catheters, subjecting them to humiliation and pain. Their rights are further violated when police coerce women into giving evidence by using their children as leverage. Street drug testing happens in other Eastern European states, for example in Georgia; the EU has issued a recommendation to stop this unlawful practice in Georgia [35]. In Estonia random street drug testing is not legal but as the study has shown it is, nevertheless, in place in the North-East area.

As in many other countries, drug policies in Estonia fail to address gender-specific needs and the circumstances of women, leaving them at risk of violations of their rights [36]. The needs of women who use drugs differs from those of their male counterparts in many respects, resulting from their societal roles as mothers and caregivers, the development of their dependence on drugs, and also from discrimination based on their status as both drug users and women [2, 37]. They also face increased stigma because of the heavier socio-economic burden from lower employment and income levels, and the lack of social support, since, in the majority of cases, such women come from a socially disadvantaged environment and/or are in relationships with partners who use drugs [2]. Consequently, women who use drugs are more prone to mental health problems and they face increased instances of violence as well as being at higher risk of contracting infectious diseases, such as HIV, through both unsafe injecting and unprotected sex [38]. The introduction of female-only services should lower the instances of human rights violations of women who use drugs in Estonia and should also decrease the risk of infectious diseases by sensibly and flexibly addressing the different gender-related drug treatment issues and should include services for mental, physical, and reproductive health [2, 39].

To summarize, the majority of the women interviewed had experienced violations of their right to health either directly, as in cases of the lack of access to drug treatment or ART, or indirectly as a result of the cumulative effect of violations of other interrelated human rights, such as the right to non-discrimination, the right to be free from ill-treatment and arbitrary detention, or the right to privacy. However, women who use drugs are excluded from the “mainstream” human and gender rights debate in Estonia. This could be explained by the very high levels of stigma around drug-related issues and the rather weak civil society. Though institutions that aim to protect human rights do exist in Estonia, they are not utilized by people who use drugs due to self-stigma, low trust in state institutions, and a lack of information; for example, none of the women interviewed knew about the existence of human rights protection mechanisms in the country.

Human rights abuses, including the denial of harm reduction services, discriminatory access to ART, punitive law enforcement practices, and coercion in the guise of treatment for drug dependence, increase the vulnerability of people who use drugs to HIV infection and negatively affect the delivery of HIV programs [40]. The protection of the rights of people who use drugs is an essential precondition to improving their health [40].

## Conclusions

Drug laws and drug enforcement practices, combined with stigma related to drugs and HIV, are the main drivers of systematic and serious violations of human rights of women who use drugs. The study has revealed numerous cases of discrimination of women who use drugs in healthcare settings, and it has also depicted barriers in access to essential services including the opioid substitution treatment, which include but are not limited to the disclosure of health-related information by health service personnel. The study findings also indicate that there is massive amount of cases of unlawful deprivation of child custody. It is apparent that through its public institutions, such as the police and child protection services, and its healthcare system, the Government of Estonia is failing in its obligations to respect, protect, and uphold the human rights of women who use drugs. Violations of human rights of women who use drugs also undermine Estonia’s efforts in HIV prevention, care, and treatment, and its overall efforts to respect, protect, and uphold the right to health. For these reasons, the Government of Estonia should address a variety of issues related to the protection of the human rights of these vulnerable members of society.

Child protection services do not exist in the vacuum where the interest of the child is the only issue they are mandated to take into account. They have to find a balance between the competing rights and interests and select interventions which protect best interests of the child with least limitations on the rights of all interested parties, including family members in particular.

The introduction of gender-sensitive harm reduction services is highly recommended in the Estonian context. Estonian should provide gender-oriented drug treatment facilities, taking into consideration the needs of women with children. To ensure the confidentiality of women’s private medical information, the Government of Estonia should consider enforcing the protection of private data in places where such cases are most widespread.

A highly punitive framework of Estonian drug policy must be changed into the supportive, human rights-centered and balanced approach to prevail over stigma and punishment. A reform of the country’s drug laws, including depanelization for drug possession, could be the right step toward decreasing violations of human rights in Estonia. Cases of police abuse and inaccessibility of legal services documented in the course of the research should also draw the attention of Estonian authorities.

## Abbreviations

ART: Antiretroviral therapy
CHALN: Canadian HIV/AIDS Legal Network
EHRA: Eurasian Harm Reduction Association
EU: European Union
HIV: Human immunodeficiency virus
OST: Opioid substitution therapy

## References

[1] Policy brief. Women who inject drugs and HIV: addressing specific needs. Vienna: UNODC, UN women, WHO, INPUD; 2014. http://www.unodc.org/documents/hiv-aids/publications/WOMEN_POLICY_BRIEF2014.pdf. Accessed 30 May 2018.

[2] Health and social responses to drug problems: a European guide. Lisbon: EMCDDA; 2017. http://www.emcdda.europa.eu/system/files/publications/6343/TI_PUBPDF_TD0117699ENN_PDFWEB_20171009153649.pdf. Accessed 30 May 2018.

[3] Benoit T, Jauffret-Roustide M (2016). Improving the management of violence experienced by women who use psychoactive substances.

[4] Malinowska-Sempruch K, Rychkova O (2014). The impact of drug policy on women.

[5] United Nations Commission on Narcotic Drugs. Joint ministerial statement of the 2014 high-level review of the commission on narcotic drugs of the implementation by member states of the political declaration and plan of action on international cooperation towards an integrated and balanced strategy to counter the world drug problem. Vienna: United Nations; 2014.

[6] Outcome Document of the (2016). United Nations general assembly special session on the world drug problem. Our joint commitment to effectively addressing and countering the world drug problem.

[7] EU Drug Strategy 2013–2020. Luxembourg: Publications Office of the European Union; 2013.

[8] EU Action Plan on Drugs 2017–2020. Luxembourg: Publications Office of the European Union; 2017.

[9] Saldanha Vinay P, Buse Kent (2016). AIDS in eastern Europe and central Asia: time to face the facts. The Lancet.

[10] Global Commission on HIV and the Law (2012). Risks, Rights and Health. UNDP.

[11] Final Report of the Regional Campaign Women Against Violence. Vilnius: EHRN; 2017. https://harmreductioneurasia.org/wp-content/uploads/2018/10/FINAL-ehrn-wav-report-eng.pdf. Accessed 30 May 2018.

[12] Declaration. Call for a Public Health oriented drug policy. Strasbourg: Committee on Social Affairs, Health and Sustain Dev, Parliamentary Assembly of the Council of Europe; 23 November 2015.

[13] Women and Drug Policy in Eurasia. https://harmreductioneurasia.org/wp-content/uploads/2018/10/Women-and-Drug-Policy.pdf-Eurasian-Harm-Reduction-Network.pdf. Accessed 30 May 2018.

[14] Penal Code of Estonia. Tallinn: Riigikogu; 2001

[15] Estonia (2018). Country Drug Report 2018. Drug laws and drug law offences.

[16] Estonia (2018). Country Drug Report 2018.

[17] Ministry of the Interior of the Russian Federation. http://bit.ly/Ministry-Internal-Affairs-Russian-Federation (2018); Judicial Department of the Supreme Court of the Russian Federation. http://www.cdep.ru/index.php?id=79 (2018). Accessed 30 May 2018.

[18] HIV in Estonia. Narrative report for global AIDS monitoring 2017. Tallinn: National Institute for health Development; 2017. https://intra.tai.ee//images/prints/documents/149847762062_HIV_in_Estonia_GAM_2017.pdf. Accessed 30 May 2018.

[19] HIV in Estonia (2016). Situation, prevention, treatment, and care. Narrative report for global AIDS response Progress reporting.

[20] Burke SE, Calabrese SK, Dovidio JF, Levina OS, Uusküla A, Niccolai LM, Abel-Ollo K, Heimer R (2015). A tale of two cities: stigma and health outcomes among people with HIV who inject drugs in St. Petersburg, Russia and Kohtla-Järve, Estonia. Soc Sci Med.

[21] Estonia Family Law Act, 2009. Tallinn: Riigikogu; 2009. https://www.riigiteataja.ee/en/eli/530102013016/consolide. Accessed 30 May 2018.

[22] Decree of the Government No. 88 of June 19, 2014 “rules for taking bio samples”. Tallinn: Riigikogu; 2014.

[23] https://www.nhs.uk/conditions/urinary-catheters/risks/. Accessed 30 May 2018

[24] Vorobjov S. HIVi levimuse ja riskikäitumise uuring Kohtla-Järve süstivate narkomaanide seas 2012. In: Uuringu kokkuvõte. [HIV and other infections and related risk behaviors among injecting drug users in Kohtla-Järve. Study report]. Tallinn: National Institute for Health Development; 2014. In Estonian. https://intra.tai.ee/images/prints/documents/139685709195_Kohtla_Jarve%20systivate%20narkomaanide%20uuring_raport.pdf. Accessed 30 May 2018.

[25] World Health Organization Regional Office for Europe. HIV/AIDS treatment and care in Estonia. Evaluation report June 2014. Copenhagen: WHO Europe; 2014. http://www.euro.who.int/en/countries/estonia/publications/hivaids-treatment-and-care-in-estonia-2014. Accessed 30 May 2018.

[26] Study on the impact of the world drug problem on the enjoyment of human rights. Report of the United Nations high commissioner for human rights. Geneva: United Nations High Commissioner for Human Rights; 2015.

[27] Kenny KS, Barrington C, Green SL (2015). I felt for a long time like everything beautiful in me had been taken out: women’s suffering, remembering, and survival following the loss of child custody. Int J Drug Policy.

[28] Csete Joanne, Kamarulzaman Adeeba, Kazatchkine Michel, Altice Frederick, Balicki Marek, Buxton Julia, Cepeda Javier, Comfort Megan, Goosby Eric, Goulão João, Hart Carl, Kerr Thomas, Lajous Alejandro Madrazo, Lewis Stephen, Martin Natasha, Mejía Daniel, Camacho Adriana, Mathieson David, Obot Isidore, Ogunrombi Adeolu, Sherman Susan, Stone Jack, Vallath Nandini, Vickerman Peter, Zábranský Tomáš, Beyrer Chris (2016). Public health and international drug policy. The Lancet.

[29] Guidelines for the psychosocially assisted pharmacological treatment of opioid dependence. Geneva: World Health Organization; 2009. 23762965

[30] EMCDDA: Drug treatment overview for Estonia. http://www.emcdda.europa.eu/countries/drug-reports/2018/estonia/treatment_en (2018). Accessed 30 May 2018.

[31] Submission to the Global Commission on HIV and Law by Kryzhevich S. Opioid substitution treatment in Belarus: limitation of patients’ rights. 2018.

[32] Act on Narcotic Drugs and Psychotropic Substances and Precursors thereof § 151(1). Tallinn: Riigikogu; 1997. https://www.riigiteataja.ee/en/eli/506052016001/consolide. Accessed 30 May 2018.

[33] Drugs, crime, and punishment—what, how much, and to whom? Presentation at the conference “Drugs, crime and punishment – where to draw the line?”, Tallinn University, 2016. https://www.just.ee/sites/www.just.ee/files/jako_salla.pdf. Accessed 30 May 2018.

[34] Criminalization of poverty as a driver of poverty in the United States. Human Rights Watch. 2017 https://www.hrw.org/news/2017/10/04/criminalization-poverty-driver-poverty-united-states. Accessed 10 Sept 2018.

[35] Report from the commission to the European Parliament and the Council. Fourth progress report on Georgia’s implementation of the action plan on visa liberalisation. 2015. {SWD(2015) 299 final} Brussels, 18.12.2015. COM (2015)684.

[36] Schleifer R, Pol L. International guidelines on human rights and drug control. A tool for securing Women’s rights in drug control policy. Health Human Rights. 2017;19(1) https://www.ncbi.nlm.nih.gov/pmc/articles/PMC5473054/. Accessed 30 May 2018. PMC5473054 28630557

[37] Csete J (2006). “Second on the needle”: human rights of women who use drugs. HIV AIDS Policy Law Rev.

[38] Azim T, Bontell I, Strathdee SA (2015). Women, drugs and HIV. Int J Drug Policy.

[39] Roberts A, Mathers B, Degenhardt L (2009). Women who inject drugs: review of their risks, experiences and needs.

[40] Jürgens R (2010). People who use drugs, HIV, and human rights. Lancet.

